# Photodegradation of Si-doped GaAs nanowire†

**DOI:** 10.1039/c9ra06365j

**Published:** 2019-12-02

**Authors:** A. C. S. Pimenta, H. Limborço, J. C. González, N. Cifuentes, Sérgio L. L. M. Ramos, Franklin M. Matinaga

**Affiliations:** Photonics Laboratory, Physics Department, UFMG Belo Horizonte Brazil matinaga@fisica.ufmg.br; Microscopy Centre of UFMG, UFMG Belo Horizonte Brazil; Nanodevices and Sensors Laboratory, UFMG Belo Horizonte Brazil; Centre for Nanomaterial Technology and Graphene – CTNano Belo Horizonte Brazil

## Abstract

Researching optical effects in nanowires may require a high pump intensity which under ambient conditions can degrade nanowires due to thermal oxidation. In this work we investigated the photodegradation of a single Si-doped GaAs nanowire by laser heating in air. To understand the changes that occurred on the nanowire we carried out Raman spectroscopy, scanning electron microscopy, energy dispersive X-ray spectroscopy, and photoluminescence spectroscopy in laser damaged regions as well as in non-affected ones. From Raman Stokes and anti-Stokes measurements we estimated the local temperature that the oxidation process of the nanowire (NW) surface starts at as 661 K, resulting in two new Raman modes at 200 cm^−1^ and 259 cm^−1^. Scanning electron microscopy and energy dispersive X-ray spectroscopy measurements showed a significant loss of arsenic in the oxidized regions, but no erosion of the nanowire. Micro-photoluminescence measurements showed the near-band-edge emission of GaAs along the nanowire, as well as a new emission band at 755 nm corresponding to polycrystalline β-Ga_2_O_3_ formation. Our results also indicate that neither amorphous As nor crystalline As were deposited on the surface of the nanowire. Combining different experimental techniques, this study showed the formation of polycrystalline β-Ga_2_O_3_ by oxidation of the nanowire surface and the limits for performing spectroscopic investigations on individual GaAs NWs under ambient air conditions.

## Abstract 2

In order to comprehend the photodegradation of GaAs NWs, we investigated their thermal oxidation process in air induced by laser heating in a broad local temperature range.

## Graphical abstract

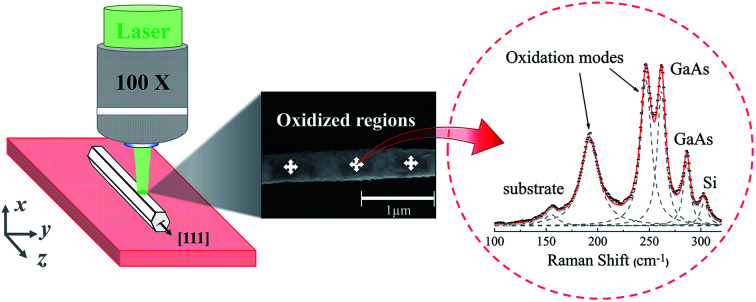

## Introduction

In recent years many studies have been devoted to the study of semiconductor nanowires (NWs).^1–4^ These nanostructures have attracted great interest due to their versatile optical^2–4^ and electrical properties,^4–6^ allowing them to be used as a building block for devices^4–9^ or even as an effective platform for an interface with biological systems.^10–12^ Thus, understanding the optical properties of these NWs is fundamental for the development of new technologies. However, some optical measurements require high pump power laser excitation,^13^ which may heat or damage the NW. Only very few works^14–18^ have focused on studying such a problem, where the heating caused by laser irradiation promoted local and irreversible damage to the NW.

In particular, thermal oxidation due to laser heating is an example of photodegradation, in which the oxidation process is accelerated by virtue of the laser incidence in an oxidizing atmosphere.^14–16^ As a consequence of this process, degradation of some features of the sample may occur, such as a change in the chemical composition due to native oxide formation;^19^ morphological modifications^16^ as in the case of surface erosion caused by loss of sample constituents;^14^ or even suppression of optical emission efficiency.^15^ Thermal oxidation in bulk GaAs has been extensively reported in the literature,^15,19–32^ and all authors agree on the formation of gallium oxides. Recently, Yazji *et al.*^15^ studied the local modifications suffered by a GaAs NW after a laser irradiation induced thermal oxidation process in air for local temperatures in the 500–600 K range. They observed that the photoluminescence emission was suppressed in the photodegraded region, but in contrast they reported a local increase of the thermal resistance of the sample. This latter conclusion was justified by the appearance of amorphous As (a-As) at low local temperatures, followed by the formation of crystalline arsenic (c-As) (*i.e.* aggregates of arsenic atoms with some periodic arrangement) as the local temperature increased. They claim that a-As and c-As were formed as thermal oxidation by-products. That work is based on a previous work by Campbell *et al.*,^33^ in which the photodegradation of bulk GaAs caused by laser irradiation was studied by Raman and photoluminescence spectroscopy. In the latter study, the appearance of a broad Raman band at 190–260 cm^−1^ for temperatures up to 500 K was attributed to the formation of a-As. By increasing the laser power density and consequently the local temperature of the sample up to 1300 K, they observed the appearance of two new Raman modes at 190 cm^−1^ and 250 cm^−1^, attributed to the formation of c-As. Note the strong temperature difference in the appearance of the c-As Raman peaks between both studies. Theoretical works have also shown the possibility of c-As formation during the thermal oxidation process,^30^ however, beyond certain temperatures its evaporation may occur, which is expected for bulk III–V semiconductor compounds.^34^ For example, He and coworkers^14^ reported c-As formation in an InAs NW, when it was irradiated by a laser. They observed the evaporation of As from the NW with increasing laser power, inducing ablation and consequently fracture of the NW. Thus, although there is a consensus about native oxide formation as by-products,^35^ some aspects related to the oxidation of GaAs NWs in air, the formation of a-As, c-As and their accumulation on the GaAs NW surface during laser heating need further investigation.

In order to comprehend the photodegradation of GaAs NWs, we investigated their thermal oxidation process in air induced by laser heating in a broad local temperature range. As will be shown, the increase of the laser beam power promoted heating of a single NW leading to local oxidation above 661 K (388 °C). Scanning electron microscopy (SEM) and energy-dispersive X-ray spectroscopy (EDS) were used to investigate morphological and chemical changes in photodegraded regions of the NW. Moreover, we examined by micro-Raman (μ-RS) and micro-photoluminescence (μ-PL) spectroscopic mapping two regions of the NW at different stages of oxidation. Based on our studies of the structural, morphological, chemical and electronic properties of the NW we show that the NW suffered photodegradation caused by a large reduction of its arsenic content and by gallium oxide formation. However, indications of the formation of amorphous or even crystalline As as oxidation by-products were not found.

## Experimental details

Free standing Si-doped GaAs nanowires (NWs) were grown on a silicon substrate by molecular beam epitaxy in a Riber 2300 R&D system,^2,36^ by the vapor–liquid–solid mechanism assisted by Au-nanoparticles. The nominal silicon concentration was 1.0 × 10^16^ cm^−3^. To study a single NW, the NWs were mechanically removed and transferred onto another Si substrate coated with a 300 nm thick layer of SiO_2_. These GaAs nanowires exhibit polytypism, which is the coexistence of alternating segments of zinc blende (ZB) and wurtzite (WZ) phases along the NW axis.^2,4,13,36,37^

In order to investigate the thermal oxidation process, we carried out μ-RS in backscattering geometry by using a WITec confocal Raman spectroscopy system equipped with both piezoelectric and motorized scanning stages. These measurements were executed at three nearby spots on the NW, separated by approximately 700 nm, at room temperature and in contact with air. A 532 nm laser line (continuous wave – CW) was focused on the sample with a 100× objective lens with a numerical aperture of 0.90. Considering Porto’s notation,^38^ the incoming light *ε*_i_ was polarized parallel to the growth axis of the NW ([0001] for WZ segments and [111] for ZB ones) and the polarization of the scattered light *ε*_s_ was not analyzed. Stokes and anti-Stokes measurements were performed with accumulation of 10 minutes and increasing the pump power in discrete steps.

Following this characterization, the morphological and chemical composition of the NW were studied by SEM and EDS. These measurements were executed in a FEI Dual Beam Quanta 3D FEG system at 20 kV high voltage. EDS and SEM were carried out in the photodegraded areas as well as in the non-degraded ones.

In sequence, a fourth nearby (5 μm away) spot on the NW was oxidized under the same conditions, thereby providing two different regions on the NW with different stages of oxidation. The second region (fourth spot) was just oxidized, while the first region (first three spots) was oxidized and subsequently annealed at a lower temperature (by conduction) during the oxidation of the second region. For a more complete characterization we acquired low-pump power μ-RS maps of non-oxidized and both thermally oxidized regions of the NW. Moreover, to investigate the optical emission properties of the NW and of the thermal oxidation by-products we performed low-temperature μ-PL measurements using a Linkan THMSE600 liquid nitrogen cryostat to stabilize the temperature at 90 K. The same WITec system was used for μ-PL mapping, but by using a 475 nm laser line and a 50× objective lens (NA 0.55).

## Results and discussion

**Fig. 1:**
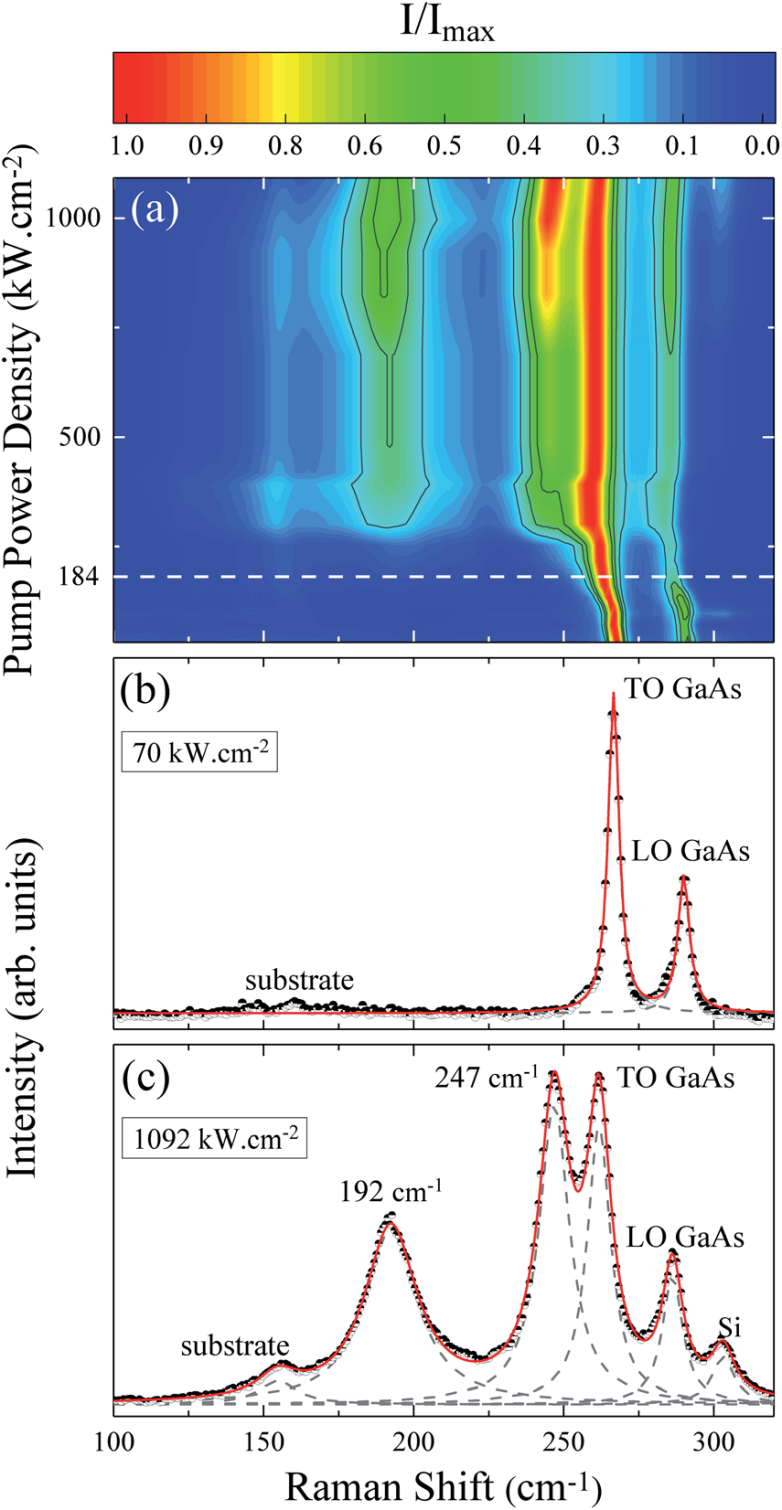
(a) Raman scattering intensities as a function of pump power density. The dashed line represents the threshold pump power density for the emergence of oxide peaks. (b) Spectrum taken at *L* = 70 kW cm^−2^. (c) Spectrum taken at *L* = 1092 kW cm^−2^. The dots are the experimental measurements, while the dashed Lorentzians represent fitting of the individual mode contributions and the red line is the total fitting curve.

The evolution of the Raman spectrum as a function of pump power density is presented in the map of Fig. 1(a). This map is composed only of Stokes peaks, and their intensities were normalized by the maximum for each spectrum. At low pump power density we can see the longitudinal optical *ν*_LO,ZB_ = 291 cm^−1^ and transverse optical *ν*_TO,ZB_ = 267 cm^−1^ modes of the ZB phase as well as the *ν*_TO,WZ_ = 267 cm^−1^ (E^H^_1_) mode of the WZ phase, see Fig. 1(b). However, as the pump power density increases the peaks shift due to the local heating of the NW. Above 184 kW cm^−2^, two new peaks appear around 200 cm^−1^ and 259 cm^−1^ (Fig. 2 of ESI†). The arising of these new modes reveals an irreversible change in the NW nature, since these modes are related to the photodegradation process (Fig. 1 of ESI†). These new modes were firstly reported in 1977 by Cape *et al.* after the thermal oxidation of bulk GaAs,^29^ nevertheless, they did not identify the origin of these peaks. Based on the literature and considering the reported chemical reactions for the thermal oxidation of GaAs,^30^ the additional Raman modes could be either associated to the *A*_3g_ (199 cm^−1^) mode of Ga_2_O_3_ (ref. 39 and 40) and the *A*_1_ (260 cm^−1^) mode of GaAsO_4_ (ref. 41) or to the TO_As_ (198 cm^−1^) and LO_As_ (257 cm^−1^) modes of c-As.^31^ There is a consensus about gallium oxide formation,^26,30,32,42^ however, some authors^15,31,33^ assign these additional Raman modes to crystalline arsenic (c-As).

Analyzing the Raman map of Fig. 1(a) again, it is possible to observe that the relative intensity of the oxide peaks increases significantly with the pump power density. The positions of these two peaks also downshift with the pump power density. These effects are clearly shown in Fig. 1(c), associated with the highest value of pump power density, corresponding to 1092 kW cm^−2^. In this spectrum, besides the GaAs modes and the oxide ones (200 cm^−1^ and 259 cm^−1^), we can see a silicon mode from the substrate at approximately 300 cm^−1^, and another substrate peak at 154 cm^−1^. These last two modes were verified through measurements done on clean substrate areas and they are not detected for low pump power conditions. In the Raman scattering theory the peak intensities are calculated from the differential spectral cross section, which in turn is related to the amount of sample which contributes to the detected scattered light.^43^ Hence, the increase in normalized intensity of the oxide peaks suggests that related by-products are being formed continuously as the pump power density increases and are accumulating on the GaAs NW. Note that the whole spectrum is well fitted by a combination of Lorentzian lines centered at the position of the above mentioned modes. An a-As mode^15,33^ at 220 cm^−1^ was not necessary. The ascription of a 220 cm^−1^ mode to a-As was previously attributed to the formation of c-As during the oxidation of GaAs by laser heating.^15,33^

The local temperature (*T*) of the NW was calculated, as a function of pump power density (*L*), by using the ratio between the Stokes (*I*_S_) and anti-Stokes (*I*_aS_) intensities of the GaAs TO mode:^44^
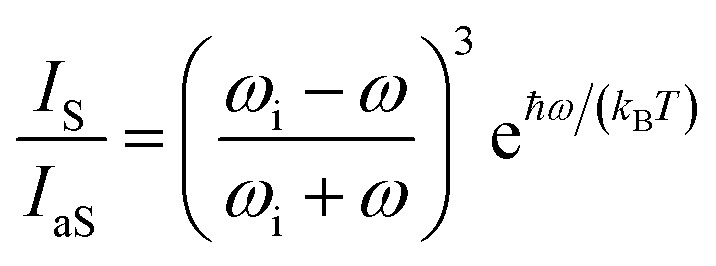
where *ω*_i_ represents the laser frequency, *ω* is the frequency of the TO GaAs mode and *k*_B_ is Boltzmann’s constant.

**Fig. 2:**
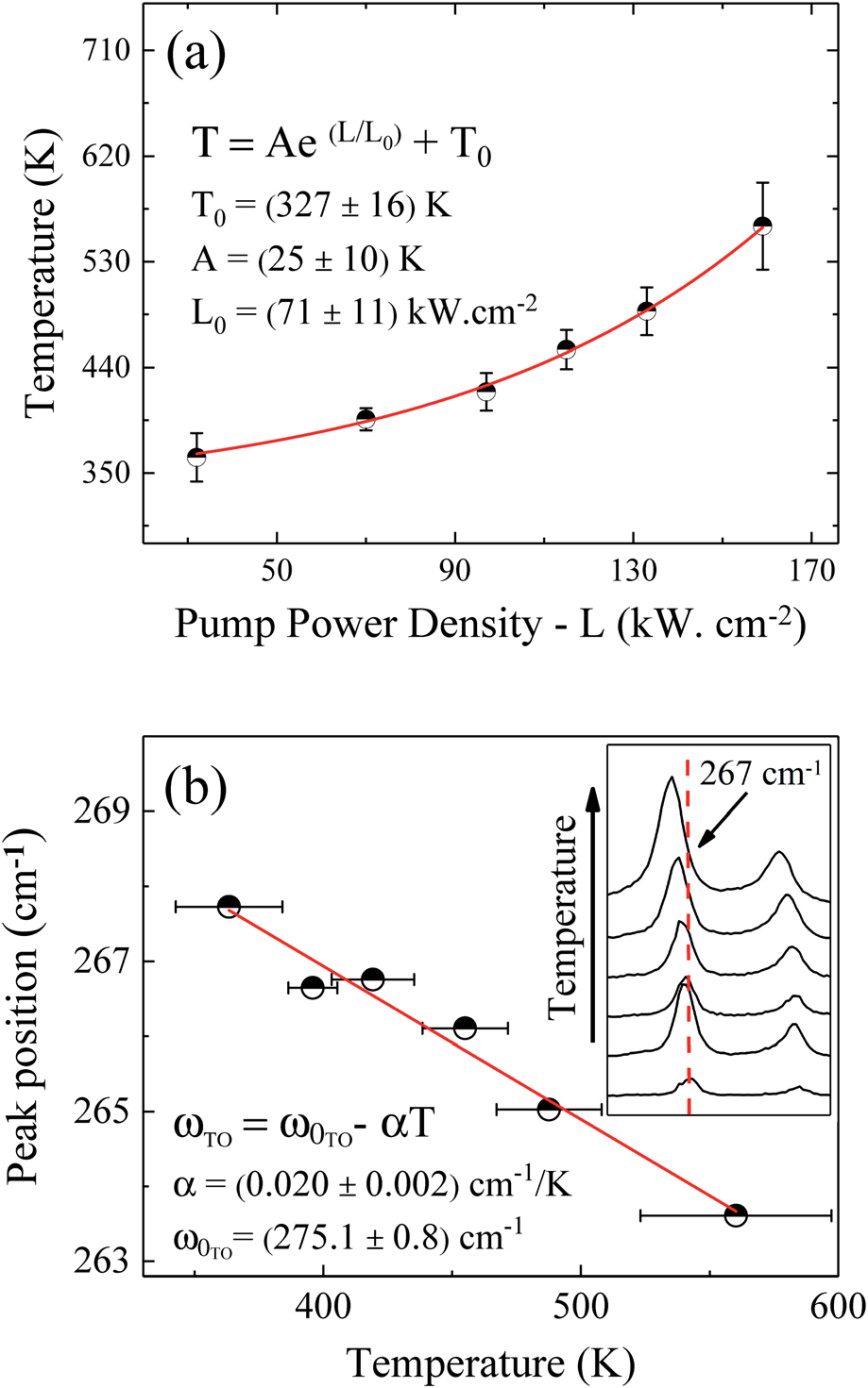
(a) Pump power density dependence of the GaAs NW local temperature. (b) Temperature dependence of the GaAs TO peak position. The inset of (b) shows explicitly the downshift of the Stokes peak.

The pump power dependence of the local temperature, as well as the downshift of the GaAs NW TO mode with temperature are shown in Fig. 2. Since the above relation is no longer valid after oxidation,^43^ we only used the data prior to this process, below *L* = 184 kW cm^−2^. Thus, this value of *L* represents a threshold temperature *T*_limit_ = 661 K (388 °C) for the start of the oxidation process. This value of the photodegradation temperature is close to the value between 500–600 K reported by Yazji *et al.*^15^ and the one reported by Persson *et al.*,^45^ who observed that the NWs suffered continuous decomposition for temperatures above 673 K (400 °C).

Laser radiation is the main factor responsible for raising the local temperature. The dependence of the local NW temperature on *L* is related to the thermal conductivity of GaAs as well as to the fraction of absorbed energy released as heat by the NW; furthermore, other conditions such as light penetration depth and width of the focused beam should also be considered.^44^ In addition to laser incidence, the contact with the environment and the poor heat dissipation between the NW and the Si substrate also contribute to the rise in local temperature and consequently, to the oxidation process. The thermal effects caused by the laser beam also promote the shift of Raman modes, which can be roughly explained by the thermal expansion of the crystal lattice.^46^ Analyzing graph 2(b) which shows the Raman temperature as a function of the GaAs TO mode (*ν*_LO,GaAs_ = 267 cm^−1^), we can explore the customary linear behavior between the increase of temperature and downshift of the peak, *ω* = *ω*_0_ − *αT*. From a linear fit we can extract the temperature coefficient *α*, whose value of 0.020 cm^−1^ K^−1^ is in agreement with the one reported for bulk GaAs (0.016 cm^−1^ K^−1^).^47^ Thus, we can ensure that the local temperatures obtained from this method reproduce reliable values.

In accordance with the Ga–As–O equilibrium phase diagram,^30^ some reactions can occur depending on the oxidation conditions, *i.e.* the temperature and surroundings in which the process takes place. In our experiment, when the process starts, we suppose that the system is in the “weak” oxidizing condition where the main product would be native gallium oxide (Ga_2_O_3_).^30^ For temperatures up to 673 K (400 °C), gallium oxide is in the amorphous phase. However, its stable polycrystalline phase (β-Ga_2_O_3_) is reached in the range of 773–973 K (500–700 °C).^24^ Arsenic oxide formation (As_2_O_3_) could be also expected to occur temporally during the oxidation process, since it is volatile above 623 K (350 °C).^26,35^

Throughout our experiments the temperature was not kept constant. Ours is a dynamical process in which other compounds could also be produced. Then, for temperatures above 773 K (500 °C), it may be possible to detect GaAsO_4_, due to the fact that some As atoms can react with Ga_2_O_3_ in these conditions.^19,24,26^ Thus, as the pump power increases and, consequently, the temperature too, we would probably achieve “intermediate” oxidizing conditions or even “stronger” ones.^30^ However, only two oxide-related peaks were observed during the whole process. These peaks were observed for temperatures above 661 K (388 °C), for which arsenic oxides are already volatile. This is a clear indication that new oxide species or a-As were not formed after the initial species at 661 K.

**Fig. 3:**
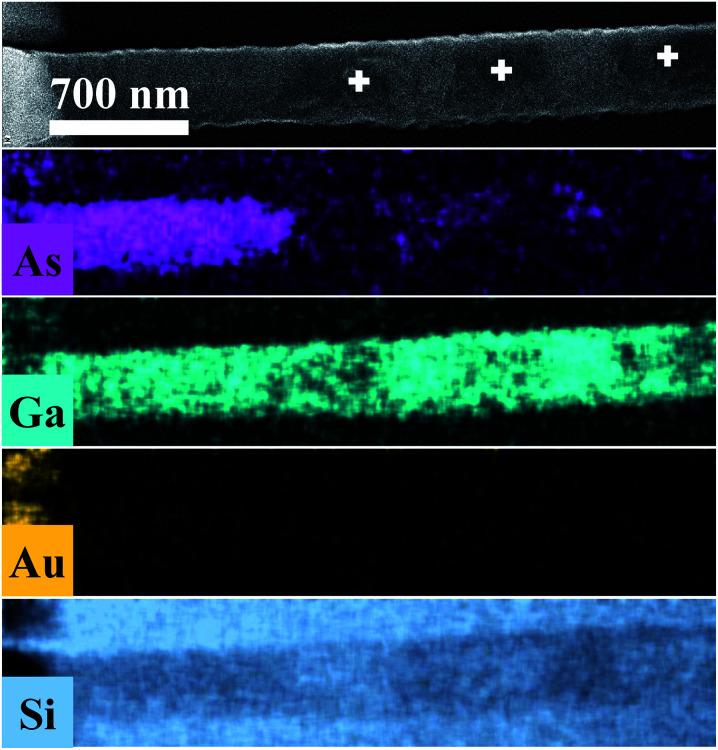
As, Ga, Au and Si EDS elemental maps for the oxidized regions of the NW. In the uppermost SEM image of the NW, the three markings show the photodegraded areas.

Fig. 3 displays As, Ga, Au and Si EDS elemental maps for the first oxidized region of the NW, including a SEM image of the same region. The EDS maps were obtained from As-Lα, Ga-Lα, Au-Lα, and Si-Kα X-Ray emission lines. The SEM image (the topmost image in Fig. 3) shows the gold contact on the left side of the NW. It is possible to see a strong contrast between the pristine regions and the oxidized ones. The three oxidized spots (dark spots marked by a white +) have diameters of about 500 nm, which corresponds to the laser spot size. These contrast variations in the SEM image are strong evidences that the sample suffered degradation, although we did not observe any decreasing of the diameter or erosion of the NW, as has been described by some researchers in similar experiments.^14,15^ A slight decrease of the Ga peak counts, as well as a strong decrease of the As peak counts, can be observed in the EDS maps of the irradiated regions. A semi-quantitative analysis of the Ga and As atomic concentrations along the NW is shown in Fig. 3 and 4 of the ESI.† From these analyses we observed a 20% reduction in the amount of As in the irradiated regions. The qualitative analysis of the oxygen concentration, shown in Fig. 3 and 4 of ESI,† also demonstrates a significant increase of oxygen in the same regions. According to the literature in “weak” oxidizing conditions,^30^ production of crystalline arsenic is expected in this process. However, rich regions of As (corresponding to a-As or even c-As agglomerations) were not found in our measurements. This observation is in agreement with the lack of observation of the a-As Raman mode, and indicates the fast evaporation of As and related oxides during the heating process.

**Fig. 4:**
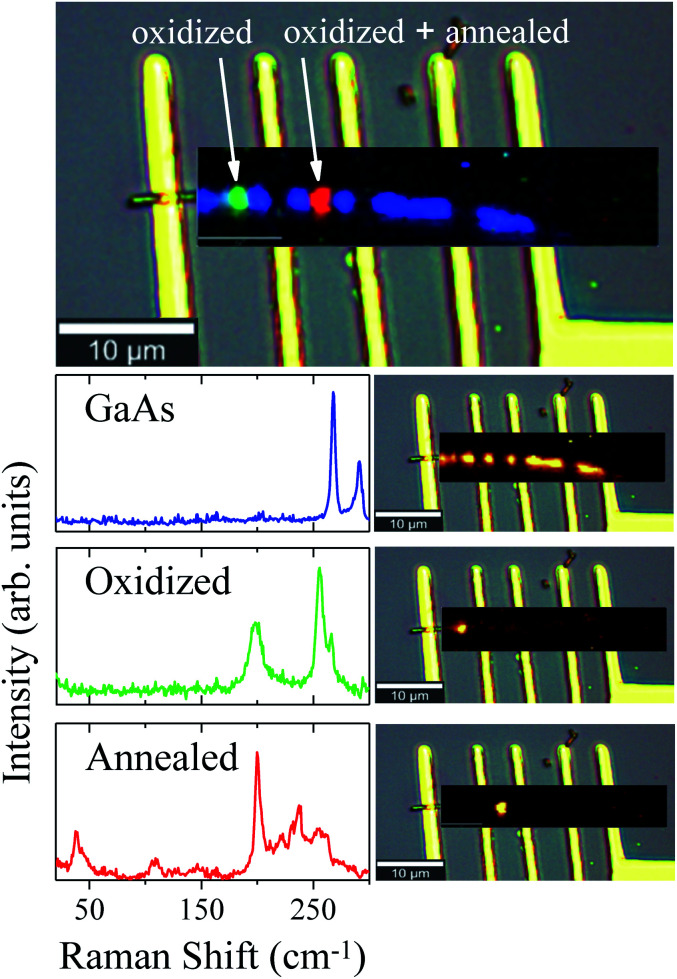
Combined spectral component distribution map of the Raman hyperspectral (topmost image) and, in sequence, the separate distributions maps, together with their respective spectra (left side) for each component after the deconvolution analysis.

The oxidation of the second region promoted different stages of oxidation in the NW, since the first region was annealed (by conduction) during the oxidation of the second one. These two different stages were investigated and verified by means of μ-RS and μ-PL mappings along the NW. Fig. 4 shows low-pump Raman maps of the first and second regions of the NW. A deconvolution analysis was applied to retrieve the main spectral components of the Raman maps, constituting the entire Raman hyperspectral. This procedure resulted in three different spectra presented in Fig. 4. The first spectrum (blue) reproduces the non-oxidized region, where we have only GaAs modes, as expected. From its corresponding map (on the right side), which shows how this spectral component is spatially distributed upon spectral deconvolution, we observe that an intense signal is present for most of the NW extension. However, in the photodegraded areas the GaAs modes are observed to be strongly reduced. The green and red Raman spectra represent the two differently oxidized regions. Examining the green spectrum, of the second oxidized region, we observe peaks at 200 cm^−1^ and 259 cm^−1^ which are associated to expected oxidation products. The corresponding map (on the right side) shows this component in a specific region of the NW. Differently, the red spectrum of the first region (oxidized and also annealed) shows low-frequency Raman modes at 39 cm^−1^ and 109 cm^−1^, besides those mentioned above, indicating some modification of the initial oxides. Since this region was heated again after oxidation, it is expected that the sample is in another stage of oxidation – it is in a higher degradation state.

**Fig. 5:**
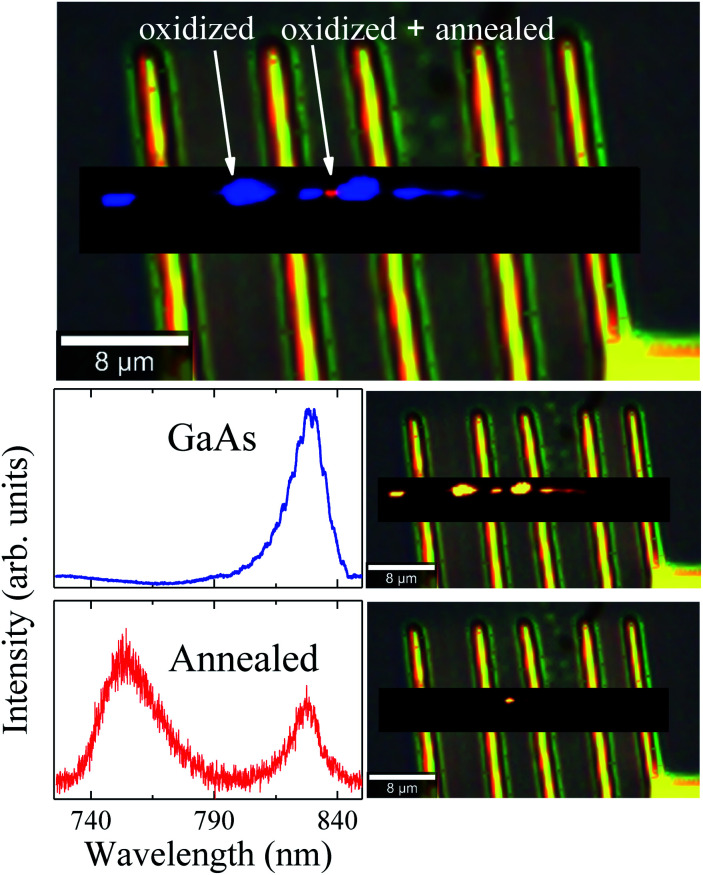
Combined spectral component distribution map for the μ-PL hyperspectral (topmost image) and, in sequence, the individual maps, together with their respective spectra (on the left side), for each spectral component after the deconvolution analysis.

To further understand the photodegradation suffered by the NW, μ-PL mappings were measured at 90 K. We obtained two different spectral components from the deconvolution procedure, as can be observed in Fig. 5. The blue spectrum depicts near-band-edge GaAs photoluminescence with a maximum around 830 nm. The combined component distribution map (on the right side of the spectrum) shows GaAs emission along the whole NW, less intense in the oxidized areas. PL peaks associated to Ga_2_O_3_ and GaAsO_4_ would not be observed in oxidized regions, since the bandgaps of these compounds^48,49^ are too large to be excited with the 475 nm laser line used in our measurements. On the other hand, a strong PL emission at 755 nm was observed in the annealed region of the NW (red spot, Fig. 5). Cheng *et al.*^50^ have observed that two new emissions at 330 nm and 706 nm appeared when the β-Ga_2_O_3_ was annealed, associated to defect levels transitions, generated during this process. Under these considerations, we suggest that the gallium oxide amorphous phase was formed above *T*_limit_ and transformed to the β-Ga_2_O_3_ polycrystalline phase as the local temperature increased. Finally, during the annealing of the already oxidized region, defect levels were generated within the β-Ga_2_O_3_ bandgap, producing the emission observed at 755 nm. Therefore, the μ-PL measurements support the formation of this kind of gallium oxide. Moreover, this outcome is evidence that the region achieved a local temperature above 773 K (500 °C), which is similar to the formation temperature of β-Ga_2_O_3_.^51^ Finnie *et al.*^52^ have shown that microcrystalline arsenic oxides, as well as bulk As_2_O_3_ and As_2_O_5_, exhibit strong PL emission at 548 nm. However, emission at this wavelength was not observed in our case. This last result is also in agreement with the μ-RS and EDS measurements, where evidences of amorphous arsenic, c-As or arsenic oxides were not observed.

## Conclusions

In summary it has been shown that under ambient conditions GaAs NWs suffer photodegradation for a laser pump power density above 184 kW cm^−2^, which corresponds to a local temperature above 661 K (388 °C). Furthermore, above this temperature we verified two new modes at 200 cm^−1^ and 259 cm^−1^ in the Raman spectra, which were related to the thermal oxidation process. Some researchers^15,31,33^ have attributed these two Raman modes to crystalline arsenic. However, we verified the absence of amorphous As Raman modes at 220 cm^−1^, the large reduction of the amount of As in the laser irradiated regions, and the absence of the characteristic green PL emission of arsenic oxides at 548 nm in our experiments. Thus, our results indicate that neither amorphous As, nor crystalline As, were deposited and oxidized on the surface of the GaAs NW during the laser heating and oxidation process. Furthermore, our measurements provide strong evidence for gallium oxide formation in its β-phase. Therefore, we ascribe the observed additional Raman modes at 200 cm^−1^ to this oxide. We conclude that the photodegradation of GaAs NWs by strong laser irradiation (up to 1092 kW cm^−2^) does not affect significantly the morphology of the NW, but results in polycrystalline gallium oxide formation, severe arsenic loss and reduction of the near-band-edge photoluminescence emission only in the oxidized and further annealed regions. Lastly, by combining different experimental techniques, our study clearly shows the limits under which spectroscopic investigations on individual GaAs NWs can be performed under ambient air conditions in terms of laser power density and local temperature.

## Conflicts of interest

There are no conflicts of interest to declare.

## Supplementary Material

RA-009-C9RA06365J-s001

## References

[cit1] Lu J., Liu H., Zhang X., Sow C. H. (2018). Nanoscale.

[cit2] Sedrine N. B., Ribeiro-Andrade R., Gustafsson A., Soares M., Bourgard J., Teixeira J., Salomé P., Correia M., Moreira M., De Oliveira A. (2018). et al.. Nanoscale.

[cit3] Falcão B., Leitão J., Correia M., Soares M., Morales F., Mánuel J., Garcia R., Gustafsson A., Moreira M., De Oliveira A. (2013). et al.. J. Appl. Phys..

[cit4] Kannappan P., Sedrine N. B., Teixeira J. P., Soares M. R., Falcão B. P., Correia M. R., Cifuentes N., Viana E. R., Moreira M. V., Ribeiro G. M. (2017). et al.. Beilstein J. Nanotechnol..

[cit5] Cifuentes N., Limborço H., Viana E., Roa D., Abelenda A., da Silva M., Moreira M., Ribeiro G., de Oliveira A., González J. C. (2016). Phys. Status Solidi B.

[cit6] Cifuentes N., Viana E., Limborço H., Roa D., Abelenda A., da Silva M., Moreira M., Ribeiro G., de Oliveira A., González J. C. (2016). J. Nanomater..

[cit7] Baxter J. B., Aydil E. S. (2005). Appl. Phys. Lett..

[cit8] Hua B., Lin Q., Zhang Q., Fan Z. (2013). Nanoscale.

[cit9] Yan J., Chen Y., Wang X., Fu Y., Wang J., Sun J., Dai G., Tao S., Gao Y. (2019). Nanoscale.

[cit10] Cui Y., Wei Q., Park H., Lieber C. M. (2001). Science.

[cit11] Wang S., Xu L.-P., Liang H.-W., Yu S.-H., Wen Y., Wang S., Zhang X. (2015). Nanoscale.

[cit12] Su S., He Y., Song S., Li D., Wang L., Fan C., Lee S.-T. (2010). Nanoscale.

[cit13] Pimenta A. C. S., Teles Ferreira D., Roa D., Moreira M., De Oliveira A., González J. C., De Giorgi M., Sanvitto D., Matinaga F. M. (2016). J. Phys. Chem. C.

[cit14] He J., Chen P., Lu W., Dai N., Zhu D.-M. (2014). Appl. Phys. A: Mater. Sci. Process..

[cit15] Yazji S., Zardo I., Soini M., Postorino P., Morral A. F. i., Abstreiter G. (2011). Nanotechnology.

[cit16] Gupta V. K., Ingale A. A., Jain V., Aggarwal R., Pal S. (2018). J. Alloys Compd..

[cit17] Tanta R., Madsen M., Liao Z., Krogstrup P., Vosch T., Nygård J., Jespersen T. S. (2015). Appl. Phys. Lett..

[cit18] Tanta R., Kanne T., Amaduzzi F., Liao Z., Madsen M. H., Alarcón-Lladó E., Krogstrup P., Johnson E., Morral A. F. i., Vosch T. (2016). et al.. Nanotechnology.

[cit19] Wilmsen C. (1981). J. Vac. Sci. Technol..

[cit20] Murarka S. (1975). Appl. Phys. Lett..

[cit21] Rubenstein M. (1966). J. Electrochem. Soc..

[cit22] Minden H. T. (1962). J. Electrochem. Soc..

[cit23] Pakes A., Skeldon P., Thompson G., Hussey R., Moisa S., Sproule G., Landheer D., Graham M. (2002). Surf. Interface Anal..

[cit24] Monteiro O. R., Evans J. W. (1989). J. Vac. Sci. Technol., A.

[cit25] Schwartz G., Gualtieri G., Griffiths J., Thurmond C., Schwartz B. (1980). J. Electrochem. Soc..

[cit26] Hollinger G., Skheyta-Kabbani R., Gendry M. (1994). Phys. Rev. B: Condens. Matter Mater. Phys..

[cit27] Levinsohn N., Beserman R., Cytermann C., Brener R., Khait Y. L., Regel G., Musolf J., Weyers M., Brauers A., Balk P. (1990). Appl. Phys. Lett..

[cit28] Koshiga F., Sugano T. (1977). Jpn. J. Appl. Phys..

[cit29] Cape J., Tennant W., Hale L. (1977). J. Vac. Sci. Technol..

[cit30] Thurmond C., Schwartz G., Kammlott G., Schwartz B. (1980). J. Electrochem. Soc..

[cit31] Farrow R. L., Chang R. K., Mroczkowski S., Pollak F. H. (1977). Appl. Phys. Lett..

[cit32] Sands T., Washburn J., Gronsky R. (1985). Mater. Lett..

[cit33] Campbell I., Fauchet P. (1990). Appl. Phys. Lett..

[cit34] Pearton S. J. , Abernathy C. R. and Ren F., Topics in growth and device processing of III-V semiconductors, World scientific, 1996, vol. 1

[cit35] Contour J., Massies J., Fronius H., Ploog K. (1988). Jpn. J. Appl. Phys..

[cit36] Falcão B., Leitão J., González J. C., Correia M., Zayas-Bazán K., Matinaga F. M., Moreira M., Leite C., De Oliveira A. (2013). J. Mater. Sci..

[cit37] Falcao B. P., Leitão J. P., Correia M. R., Leitão M. F., Soares M. R., Moreira M. V., de Oliveira A. G., Matinaga F. M., González J. C. (2014). J. Mater. Chem. C.

[cit38] Zardo I., Conesa-Boj S., Peiro F., Morante J., Arbiol J., Uccelli E., Abstreiter G., Morral A. F. i. (2009). Phys. Rev. B: Condens. Matter Mater. Phys..

[cit39] Kranert C., Sturm C., Schmidt-Grund R., Grundmann M. (2016). Sci. Rep..

[cit40] Pearton S., Yang J., Cary IV P. H., Ren F., Kim J., Tadjer M. J., Mastro M. A. (2018). Appl. Phys. Rev..

[cit41] Cambon O., Bhalerao G., Bourgogne D., Haines J., Hermet P., Keen D., Tucker M. (2011). J. Am. Chem. Soc..

[cit42] Grovenor C., Cerezo A. (1989). J. Appl. Phys..

[cit43] Hayes W. and Loudon R., Scattering of light by crystals, Courier Corporation, 2012

[cit44] Pelletier M. J. , Analytical applications of Raman spectroscopy, Wiley-Blackwell, 1999

[cit45] Persson A. I., Larsson M. W., Stenström S., Ohlsson B. J., Samuelson L., Wallenberg L. R. (2004). Nat. Mater..

[cit46] Han L., Zeman M., Smets A. H. (2015). Nanoscale.

[cit47] Besson J., Itie J., Polian A., Weill G., Mansot J., Gonzalez J. (1991). Phys. Rev. B: Condens. Matter Mater. Phys..

[cit48] Li L., Auer E., Liao M., Fang X., Zhai T., Gautam U. K., Lugstein A., Koide Y., Bando Y., Golberg D. (2011). Nanoscale.

[cit49] Christie D. M., Chelikowsky J. R. (1998). J. Phys. Chem. Solids.

[cit50] Cheng Y., Chen J., Yang K., Wang Y., Yin Y., Liang H., Du G. (2014). J. Vac. Sci. Technol., B.

[cit51] Stepanov S., Nikolaev V., Bougrov V., Romanov A. (2016). Rev. Adv. Mater. Sci..

[cit52] Finnie C. M., Bohn P. W. (1999). Appl. Phys. Lett..

