# Supplementary material for: Photodegradation of Si-doped GaAs nanowire
Source: RSC Adv. 2019 Dec 2;9(67):39488–94. doi: 10.1039/c9ra06365j (PMC9076065; doi:10.1039/c9ra06365j)
Supplement: RA-009-C9RA06365J-s001 [file RA-009-C9RA06365J-s001.pdf]

Electronic Supplementary Material (ESI) for RSC Advances.
This journal is © The Royal Society of Chemistry 2019

Electronic Supplementary Information: Photodegradation of Si-doped GaAs Nanowire

Ana Clara Sampaio Pimenta and Franklin Massami Matinaga

Photonic Laboratory, UFMG, Belo Horizonte, Brazil

Henrique Limborço

Centre of Microscopy of UFMG, Belo Horizonte, Brazil

Juan Carlos González Pérez and Nestor Cifuentes Taborda

Nanodevices and Sensors Laboratory, UFMG, Belo Horizonte, Brazil

Sérgio Luís Lima de Moraes Ramos

Centre for Nanomaterials technology and Graphene - CTNano, Belo Horizonte, Brazil

(Dated: 2019)

PHOTODEGRADATION PROCESS

Irreversibility of the process

In order to confirm the irreversibility of the photodegradation, μ-Raman measurements at low pump power condition were performed after the process, at the same positions within the nanowire. The setup used was similar to the other measurements in the main work. Figure 1 shows the peaks attributed to the by-products of oxidation, even at low pump power density (L) values.

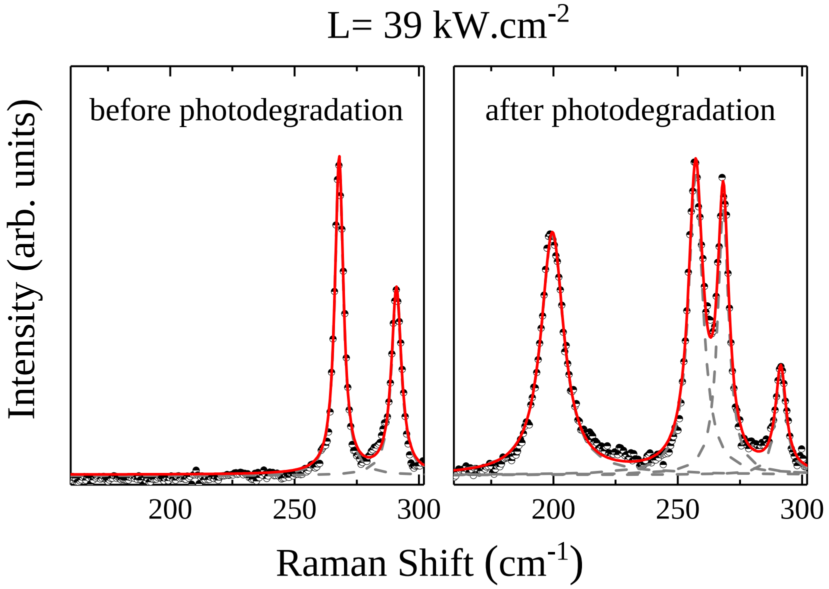

FIG. 1. Raman spectra at low pump power density before and after the photodegradation process.

Raman spectra as a function of pump power density - L

To estimate in which pump power density began the photodegradation process, we analyzed each Raman spectrum and noted that from L = 184 kW.cm^{-2} vibrational modes attributable to oxidation by-products were observed.

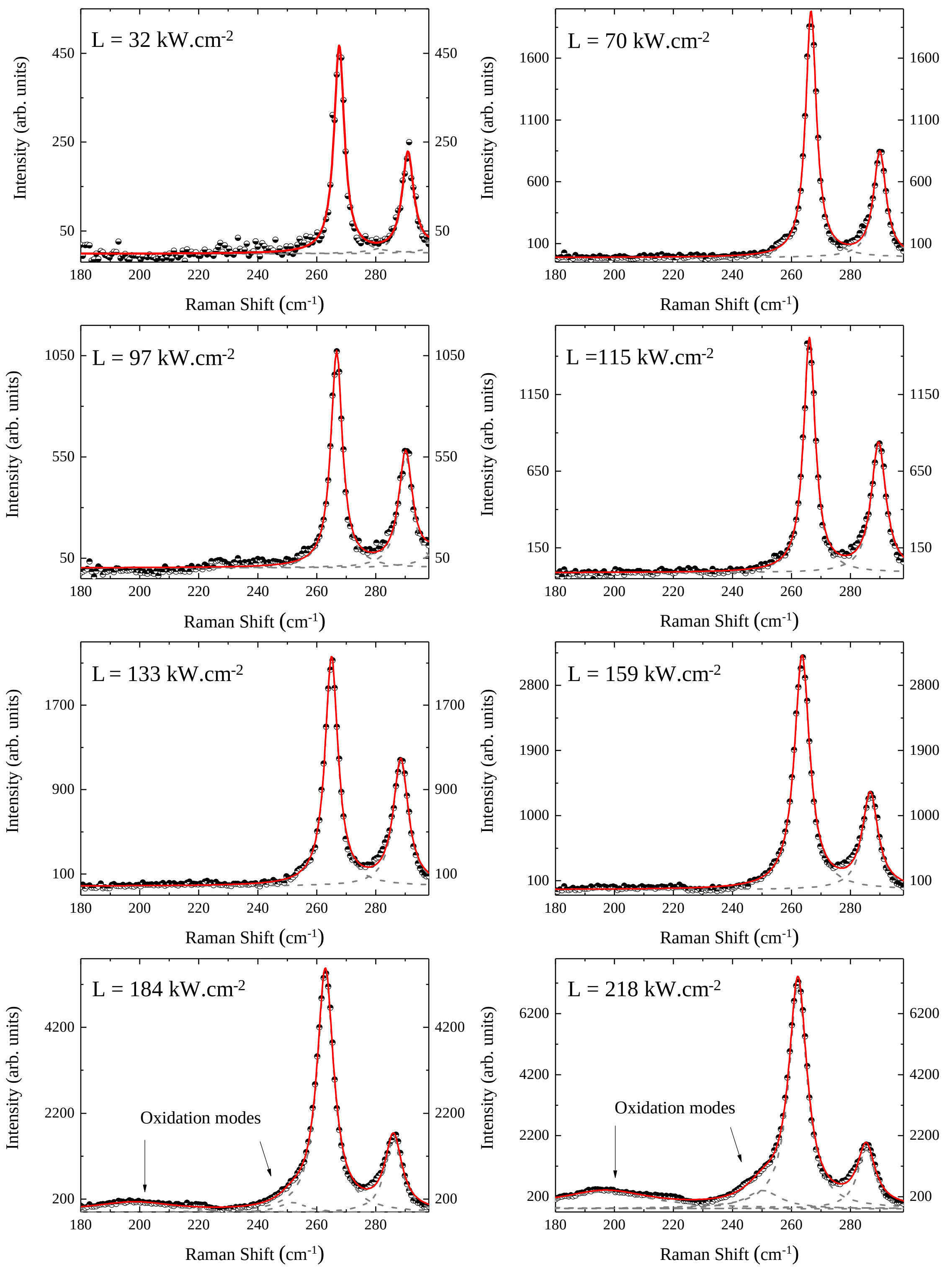

FIG. 2. Raman spectrum for each power density of the pump until the start of the photodegradation.

EDS LINESCAN MEASUREMENTS

The local chemical composition of the GaAs NW is shown in our EDS linescans measurements, Figure 3. The EDS spectra were acquired during 30 min using 20 kV high voltage, 4.5 spot size and 50 um objective aperture for effective excitation of K lines for both Ga and As, and good signal to noise ratio. Atomic % concentration was obtained using the P/B-ZAF method for both K-α and L-α X-ray emissions taking as reference the GaAs stoichiometric composition in the non-affected region of the NW. Figure 3 (a) shows quantified EDS linescan along a non-affected region, where small fluctuations from the GaAs stoichiometry (1%) are within the error margin for the technique. Analyzing the EDS linescan of Figure 3 (b), on the other hand, we can observe a large decrease in atomic % for As in the laser-affected regions, which confirms the loss of As in this area. This graph also exhibits the small fluctuations of the Ga composition in the oxidized region, but it is not as relevant as the As decrease presented, Fig. 3 (b).

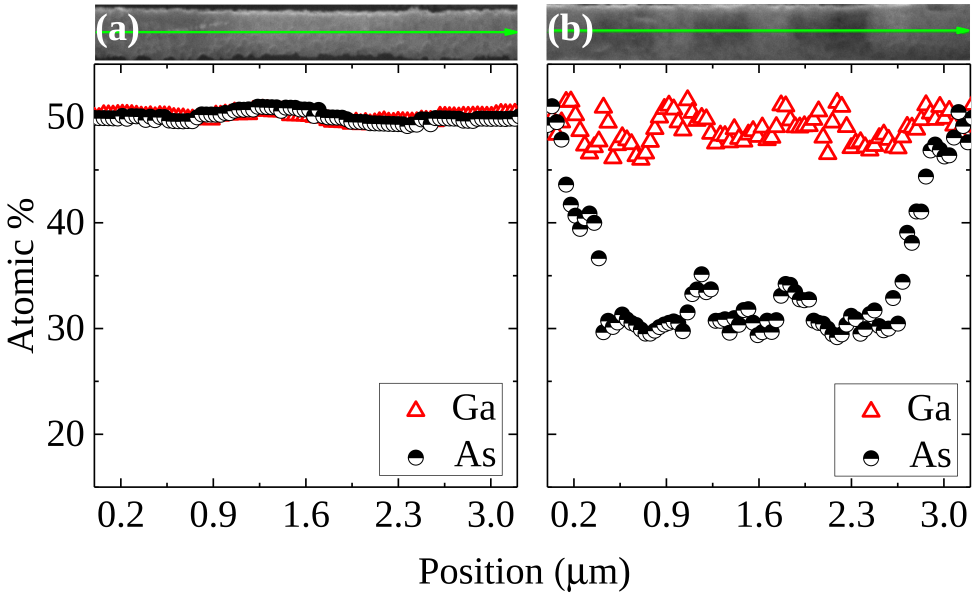

FIG. 3. **(a)** EDS linescans for As and Ga on NW region not affected by laser. The atomic concentration was calculated by P/B-ZAF method for each point spectra along the green line in secondary electrons image. **(b)** Average EDS atomic % concentration of Ga and As in the NW laser modified region. EDS line quantification was also performed with P/B-ZAF method for each point spectra (100 points) collected along the green line indicated in secondary electrons image.

From the EDS measurements for oxygen species, we observed that an absolute value cannot be obtained for the amount of oxygen in the sample because there is no known stoichiometric reference containing oxygen in the NW. However, when we compared the number of counts per second (cps) of characteristic oxygen X-rays for the non-damaged region (Fig.3 (a)) and the damaged regions by the laser (Fig.3 (b)), we realized that there was an increase in X-rays emission in the modified region. This evidence corroborates the model of Ga oxide formation in NW.

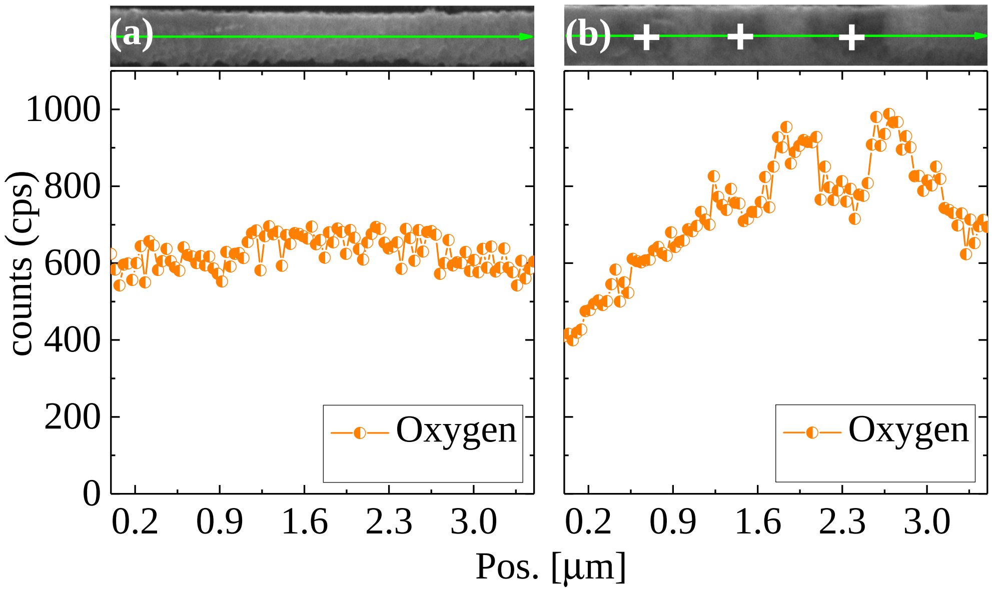

FIG. 4. **(a)** EDS linescans for oxygen in the same regions of graph 3, in the non-damaged region. The atomic concentration was calculated by P/B-ZAF method for each point spectra along the green line in secondary electrons image. **(b)** Average EDS atomic % concentration of oxygen in the NW laser modified region. EDS line quantification was also performed with P/B-ZAF method for each point spectra (100 points) collected along the green line indicated in secondary electrons image.
